# 

**Published:** 1850-10-01

**Authors:** 


### CONTENTS.

| | PAGE |
|---|---|
| The History and Present State of Lunatic Asylums—Public and Private | 425 |
| The Trial of Robert Pate, or the Plea of Lunacy | 445 |
| The Ohio Lunatic Asylum | 456 |
| Mixed Insanity—Reason and Madness | 490 |
| The Pathology of Insanity. By Dr. Hitchman | 501 |
| The Morbid Anatomy of the Coverings of the Brain. By H. Coote, Esq., F.R.C.S. | 521 |
| Notes of a Recent Visit to several Provincial Asylums for the Insane in France. By Dr. Webster, F.R.S. | 529 |
| On Tædium Vitæ. By Dr. B. de Boismont | 540 |
| Dr. Ogilvie's Course of Lectures on Medical Psychology | 557 |
| Trial of Robert Pate for the Outrage upon her Majesty | 557 |
| The State Lunatic Asylum, New York. (With an Engraving.) | 561 |
| English County Lunatic Asylums | 562 |
| Testimonial to Dr. Conolly | 565 |
| Books, &c., received | 565 |
| Periodicals in Exchange | 568 |

INDEX TO VOLUME III.

